# Do We Need to Know the Left Ventricular Geometry Patterns of the Brazilian Population?

**DOI:** 10.36660/abc.20190838

**Published:** 2020-01

**Authors:** Roberto M. Saraiva

**Affiliations:** Fundação Oswaldo Cruz - Instituto Nacional de Infectologia Evandro Chagas, Rio de Janeiro, RJ - Brazil

**Keywords:** Heart Failure, Hypertension, Hypertrophy, Left Ventricular, Ventricular Remodeling, Heart Ventricles/cirurgia, Echocardiography/métodos

In this issue of the *Arquivos Brasileiros de Cardiologia*, Almeida et al.^1^ describe the left ventricular (LV) remodeling patterns frequencies found in a Brazilian population followed at primary healthcare clinics in Niterói city, state of Rio de Janeiro. The authors found that a LV abnormal geometry was present in up to 33% of 636 studied individuals (mean age 59.5±10.3 years old; 62% women). Eccentric LV hypertrophy (LVH) was the most common abnormal LV geometry pattern (29%), followed by concentric LVH and concentric remodeling (2% each).

LV remodeling is no longer considered solely an adaptative mechanism but a response to several different stimuli that lead to gene activation, cellular hypertrophy, apoptosis, fibrosis, and, finally, LV remodeling with different degrees of LV function compromise and increase in cardiovascular risk.^2^ In fact, the relation between LVH diagnosed by electrocardiogram and mortality has been long recognized.^3^ LV mass is considered an independent risk factor for heart failure (HF),^4,5^ stroke,^5^ sudden cardiac death,^6^ supraventricular and ventricular tachycardia,^7^ and all-cause^8^ and cardiovascular mortality.^9^ Therefore, hypertension (HTN) is considered stage A HF and LVH is considered stage B HF on the American College of Cardiology/American Heart Association guidelines on HF management.^10^ Surprisingly, it was not up to the study of Almeida et al.^1^ that LV geometry patterns were studied in Brazilian population. We need to know exactly what are the frequency and value of LV geometry patterns in the Brazilian population and not only apply knowledge obtained with other populations.

Many different factors and stimuli influence LV geometry remodeling such as age, gender,^11^ severity, duration and treatment status of HTN,^12^ obesity,^13,14^ metabolic syndrome,^15^ and diabetes mellitus.^16^ Almeida et al.^1^ also showed an association between eccentric LVH and gender, age, level of education, HTN, and albumin/creatinine ratio. However, the frequencies of those factors may have a great variation between populations which shows the importance of specifically addressing the LV geometry patterns and their prognostic value in Brazilian population.

LV abnormal geometry is classified into concentric remodeling (normal LV mass with increased relative wall thickness), concentric LVH (increased LV mass and relative wall thickness), and eccentric LVH (increased LV mass and normal relative wall thickness)^17^ based on M-mode echocardiography. LV geometric abnormalities are usually found in the general population. However, the distribution of the kind of LV geometry abnormalities may vary between studies. In a study with 35,602 patients with normal LV ejection fraction referred for echocardiography, concentric remodeling was identified in 35%, concentric LVH in 6% and eccentric LVH in 5%.^8^ However, this prevalence increases with ageing. In elderly patients, concentric remodeling was found in 43%, concentric LVH in 8.5% and eccentric LVH in 7.4%.^18^ Those results are strikingly different from the data described in the Brazilian population by Almeida et al.^1^ with a higher prevalence of eccentric LVH. Such a difference may be related to the high prevalence of HTN and diabetes in the population studied by Almeida et al.^1^ In fact, the most common type of LVH in patients with HTN is eccentric and not concentric LVH.^12^ Nevertheless, such differences between studies underscore the importance of studies addressing the Brazilian population. For instance, eccentric hypertrophy was associated with the development of HF with reduced ejection fraction, while concentric LVH was associated with the development of HF with preserved ejection fraction.^19^

A new classification for LVH was proposed based on LV dilation and concentricity:^20^ concentric non-dilated, concentric dilated, eccentric non-dilated and eccentric dilated. The importance of this new classification was demonstrated by the fact that eccentric non-dilated LVH is not associated with poor outcomes while all others had increased risk of all-cause and cardiovascular disease (CVD) mortality^21^ or increased risk of HF or CVD death compared to participants without LVH.^22^

Thus, I congratulate Almeida et al.^1^ for their very important research and I challenge them to pursue on their research and present the classification of LV geometry based on 4-tiered classification of LVH and, more importantly, the prognostic value of LV remodeling patterns in Brazilian population followed at primary healthcare.
